# Antimicrobial-Resistant *Klebsiella pneumoniae* Carriage and Infection in Specialized Geriatric Care Wards Linked to Acquisition in the Referring Hospital

**DOI:** 10.1093/cid/ciy027

**Published:** 2018-01-11

**Authors:** Claire L Gorrie, Mirjana Mirceta, Ryan R Wick, Louise M Judd, Kelly L Wyres, Nicholas R Thomson, Richard A Strugnell, Nigel F Pratt, Jill S Garlick, Kerrie M Watson, Peter C Hunter, Steve A McGloughlin, Denis W Spelman, Adam W J Jenney, Kathryn E Holt

**Affiliations:** 1 Department of Biochemistry and Molecular Biology, Bio21 Molecular Science and Biotechnology Institute, Melbourne, Victoria, Australia; 2 Department of Microbiology and Immunology at the Peter Doherty Institute for Infection and Immunity, The University of Melbourne, Melbourne, Victoria, Australia; 3 Microbiology Unit, Alfred Health, Melbourne, Victoria, Australia; 4 Wellcome Trust Sanger Institute, Hinxton, Cambridgeshire, United Kingdom, Melbourne, Victoria, Australia; 5 Infectious Diseases Clinical Research Unit, The Alfred Hospital, Melbourne, Victoria, Australia; 6 Aged Care, Caulfield Hospital, Alfred Health, Melbourne, Victoria, Australia; 7 Intensive Care Unit, Melbourne, Victoria, Australia; 8 Microbiology Unit & Department of Infectious Diseases, The Alfred Hospital, Melbourne, Victoria, Australia

**Keywords:** *Klebsiella pneumoniae*, genomic epidemiology, geriatric care, multidrug resistance, asymptomatic carriage

## Abstract

**Background:**

*Klebsiella pneumoniae* is a leading cause of extended-spectrum β-lactamase (ESBL)–producing hospital-associated infections, for which elderly patients are at increased risk.

**Methods:**

We conducted a 1-year prospective cohort study, in which a third of patients admitted to 2 geriatric wards in a specialized hospital were recruited and screened for carriage of *K. pneumoniae* by microbiological culture. Clinical isolates were monitored via the hospital laboratory. Colonizing and clinical isolates were subjected to whole-genome sequencing and antimicrobial susceptibility testing.

**Results:**

*K. pneumoniae* throat carriage prevalence was 4.1%, rectal carriage 10.8%, and ESBL carriage 1.7%, and the incidence of *K. pneumoniae* infection was 1.2%. The isolates were diverse, and most patients were colonized or infected with a unique phylogenetic lineage, with no evidence of transmission in the wards. ESBL strains carried *bla*_CTX-M-15_ and belonged to clones associated with hospital-acquired ESBL infections in other countries (sequence type [ST] 29, ST323, and ST340). One also carried the carbapenemase *bla*_IMP-26_. Genomic and epidemiological data provided evidence that ESBL strains were acquired in the referring hospital. Nanopore sequencing also identified strain-to-strain transmission of a *bla*_CTX-M-15_ FIB_K_/FII_K_ plasmid in the referring hospital.

**Conclusions:**

The data suggest the major source of *K. pneumoniae* was the patient’s own gut microbiome, but ESBL strains were acquired in the referring hospital. This highlights the importance of the wider hospital network to understanding *K. pneumoniae* risk and infection prevention. Rectal screening for ESBL organisms on admission to geriatric wards could help inform patient management and infection control in such facilities.

## Teaser

Patients’ own gut microbiota were the major source of *Klebsiella pneumoniae*, but extended-spectrum β-lactamase strains were acquired in the referring hospital. This highlights the potential for rectal screening, and the importance of the wider hospital network, for local risk management.

*Klebsiella pneumoniae* is an opportunistic bacterial pathogen associated with urinary tract infections (UTIs), pneumonia, septicemia, and wound and soft-tissue infections in healthcare settings [1]. One of the ESKAPE pathogens, which are collectively responsible for the majority of hard-to-treat infections in hospitalized patients [2], *K. pneumoniae* is frequently multidrug resistant (MDR; defined as resistant to ≥3 classes of antibiotics). Of particular concern are isolates that produce extended-spectrum β-lactamases (ESBLs) or carbapenemases, which confer resistance to third-generation cephalosporins and carbapenems, respectively [2]. Among those at risk are infants (who have immature immune systems) and the elderly (who have waning immune defenses); both are subject to heightened incidence and severity of infections [3]. *K. pneumoniae,* carried asymptomatically in the gastrointestinal (GI) tract, can disseminate to cause healthcare-associated infections in at-risk individuals [4, 5]. 

We recently reported a positive association between *K. pneumoniae* GI carriage and age among patients in an intensive care unit (ICU) [4]. Older age and hospital stays in geriatric and long-term care facilities have previously been linked with MDR bacterial colonization and infection [6–8]; hence, geriatric hospital patients can be considered an at-risk group for carriage and/or infection with *K. pneumoniae*. In the current study, we aimed to investigate the prevalence, diversity and antimicrobial resistance (AMR) of *K. pneumoniae* carried in the GI and respiratory tracts of patients admitted to 2 geriatric care units.

## METHODS

### Ethics

Ethical approval for this study was granted by the Alfred Hospital (AH) Ethics Committee (project No. 550/12).

### Recruitment and Specimen and Data Collection

Adult patients aged ≥50 years were recruited from 2 geriatric medicine wards at the Caulfield Hospital (CH). Verbal consent to participate was required from the patient or an adult responsible for them. Rectal and screening swab samples were taken at recruitment, usually within the first week of admission to the ward. Information on age, sex, dates of hospital and ICU admission/s, surgery in the last 30 days, and antibiotic treatment in the last 7 days were extracted from hospital records and recorded in a questionnaire. All clinical isolates recovered from patients at CH or the referring hospital (AH) and identified as *K. pneumoniae* infections by the AH diagnostic laboratory as part of routine care were included in the study. See Supplementary Methods for further details.

### Whole-Genome Sequence Analysis

DNA was extracted and sequenced via Illumina HiSeq. Multilocus sequence typing (MLST) was conducted using SRST2 software (version 0.2.0) [9]. Single-nucleotide variants (SNVs) were called by aligning reads to a reference genome (*K. pneumoniae* strain NTUH-K2044), and used to infer maximum likelihood phylogenetic trees. Phylogenetic lineages were defined at a threshold of >0.1% divergence. Draft genome assemblies were constructed using SPAdes software (version 3.6.1) and used to identify capsule loci with Kaptive software (version 0.4) [10]. Isolates selected for finishing (n = 17) were subjected to long-read sequencing with Oxford Nanopore MinION and hybrid assembly of long and short reads using Unicycler software (version 0.4.0) [11], as described elsewhere [12]. All read sets and finished assemblies were deposited in NCBI (accession Nos. in Supplementary Table S1). See Supplementary Methods for full details.

### AMR and Plasmid Analysis

All clinical and carriage isolates were subjected to antimicrobial susceptibility testing using the Vitek2 GNS card and Clinical and Laboratory Standards Institute break points. AMR genes were identified from Illumina reads using SRST2 (version 0.2.0) [9] to screen against the ARG-Annot database [13] (Supplementary Table S1). The locations of AMR genes were confirmed by BLAST (Basic Local Alignment Search Tool) analysis of finished genome sequences, and the Repository of Antibiotic Resistance Cassettes database and annotation service [14]. Plasmid incompatibility types and subtypes were identified using PlasmidFinder [15] and established methods for IncC subtyping [16, 17].

### Statistical Analysis

All statistical analyses were conducted using R software (version 3.3.1).

## RESULTS

During the 1-year study, 296 adults aged ≥50 years and admitted to 2 geriatric wards of CH (30.5% of 973 patients admitted to these wards) were screened for *K. pneumoniae* carriage via rectal and throat swab samples. Participant characteristics are given in Table 1. Approximately half (n = 144; 49%) were male, and age distributions were similar for both sexes, with a median age of 84 years (range, 55–102 years). The median time of recruitment was day 8 of the current hospital admission (range, days 1–83). Of the 296 patients screened, 124 (42%) had received antimicrobial therapy in the last 7 days, and 34 (11%) had undergone ≥1 surgical procedure in the last 30 days (Table 1).

**Table 1.** Characteristics of Participants Screened for *Klebsiella pneumoniae* Carriage

Characteristic	Total (n = 296)	Male (n = 144)	Female (n = 152)
Age, median (range), y	86 (55–102)	85 (55–97)	86 (58–102)
Timing of baseline swab sample, median (range), d of current hospital admission^a^	8 (1–83)	7 (1–69)	9 (1–83)
Recent antibiotics, No. (%)^b^	123 (42)	59 (41)	64 (42)
Recent surgery, No. (%)^c^	34 (11)	15 (10.4)	19 (12.5)
*K. pneumoniae*–positive culture, No. (%)			
Rectal swab sample	32 (10.8)	19 (6.3)	13 (8.6)
Throat swab sample	12 (4.1)	7 (4.9)	5 (3.3)
Both	4 (1.4)	3 (2.1)	1 (0.7)

^a^The day of admission was considered day 1.

^b^“Recent antibiotics” was defined as antimicrobial therapy in the last 7 days before screening.

^c^“Recent surgery” was defined a surgical procedures of any kind in the last 30 days before screening.

### *Klebsiella* Carriage

Isolates identified as *K. pneumoniae* were cultured from 13.5% of participants (Table 1). We estimate the point prevalence of GI carriage at 10.8% (95% confidence interval [CI], 7.6%–15.1%) and throat carriage at 4.1% (2.2%–7.2%). The carriage rates were similar among male and female participants (16% and 11%, respectively; *P* = .30 using χ^2^ test). Carriage was not significantly associated with age, sex, or day of admission in logistic regression models (Supplementary Table S2), although the study was underpowered to investigate this conclusively. GI carriage of ESBL *K. pneumoniae* was detected in 5 participants (1.7%); 4 of these isolates were also MDR (Table 2).

**Table 2.** Details of Participants With Rectal Screening Swab Samples Positive for Extended-Spectrum **β**-Lactamase *Klebsiella pneumoniae*

Patient	Age, y	Sex	Recent Surgery^a^	Recent Antibiotics^b^	Isolate^c^	Antimicrobials to Which Resistance Was Detected^d^
CH0031	96	M	No	Cro	KSB1_1B	Kz, Cro
CH0138	91	F	Yes	Tmp	KSB1_9D	Amc, Tim, Tzp, Kz, Caz, Cro, Gen, Tob, Cip, Nor, Tmp, Sxt
CH0142	72	M	No	…	KSB1_4E	Tim, Tzp, Kz, Fox, Caz, Cro, Tob, Cip, Tmp, Sxt
CH0260	72	F	No	Amc	KSB1_10J	Kz, Cro, Tmp, Sxt
CH0274	73	F	No	Van, Cip, Cfx	KSB2_1B	Kz, Cro, Tmp, Sxt

Abbreviations: Amc, amoxicillin–clavulanic acid; Caz, ceftazidime; Cfx, cefuroxime; Cip, ciprofloxacin; Cro, ceftriaxone; F, female; Fox, cefoxitin; Gen, gentamicin; Kz, cefazolin; M, male; Nor, norfloxacin; Sxt, trimethoprim-sulfamethoxazole; Tim, ticarcillin–clavulanic acid; Tmp, trimethoprim; Tob, tobramycin; Tzp, tazobactam-piperacillin; Van, vancomycin.

^a^“Recent surgery” indicates surgery of any kind in the last 30 days before screening.

^b^“Recent antibiotics” were defined as antibiotic treatment in the last 7 days before screening.

^c^CH0031 isolate KSB1_1B carried *bla*_CTX-M-55_; the others carried *bla*_CTX-M-15_.

^d^Antimicrobial resistance phenotyping was conducted using Vitek2 software and interpreted using Clinical and Laboratory Standards Institute thresholds. All isolates were confirmed resistant to ampicillin. Additional drugs to which acquired resistance was detected are listed. All 5 isolates were susceptible to cefepime, meropenem, and amikacin.

### *K. pneumoniae* Infections

Twelve (1.2%) of the CH study ward patients (7 female, 5 male) had *K. pneumoniae* infections diagnosed (all UTIs). Patient characteristics are shown in Table 3. The *K. pneumoniae* UTI rate was very low in noncarriers (0.76%; 2 of 264), however, the numbers are too small for meaningful comparisons with the rate in carriers (1 of 32; 3%; note that 9 UTIs occurred in patients not screened). Four of the 14 UTI isolates (from 12 patients) were MDR, and 3 produced ESBLs (Table 3).

**Table 3.** Details of *Klebsiella pneumoniae* Urinary Tract Infections^a^

Patient	Age, y	Sex	Carriage Positive	Antimicrobials to Which Clinical Isolate Displayed Resistance^b^
CH0110	92	F	No	Isolate 1: …
				Isolate 2: Tmp and Sxt
CH0138	91	F	Yes	Isolate 1: Amc, Tim, Tzp, Kz, Caz, Cro,^c^ Gen, Tob, Cip, Nor, Tmp, Sxt
				Isolate 2: Amc, Tim, Tzp, Kz, Caz, Cro,^c^ Gen, Tob, Cip, Nor, Tmp, Sxt, Fox, Fep, Mer
CH0258	91	F	No	Amc, Tim, Kz, Caz, Cro,^c^ Tob, Tmp, Sxt
KC0049	78	F	NP	…
KC0061	90	F	NP	…
KC0109	69	M	NP	…
KC0191	91	M	NP	Amc, Tim, Tzp, Kz, Cro,^c^ Fep, Tob, Tmp, Sxt
KC0216	86	M	NP	Nor
KC0302	81	M	NP	…
KC0303	71	M	NP	…

Where two clinical isolates were cultured, these are labelled 1 and 2, and antimicrobial resistance phenotypes are given for both.

Abbreviations: Amc, amoxicillin–clavulanic acid; Ami, amikacin; Caz, ceftazidime; Cip, ciprofloxacin; Cro, ceftriaxone; F, female; Fep, cefepime; Fox, cefoxitin; Gen, gentamicin; Kz, cefazolin; M, male; Mer, meropenem; Nor, norfloxacin; NP, not participating; Sxt, trimethoprim-sulfamethoxazole; Tim, ticarcillin–clavulanic acid; Tmp, trimethoprim; Tob, tobramycin; Tzp, tazobactam-piperacillin.

^a^Characteristics of patients and corresponding urine isolates are provided. Carriage screening swab sample results are given for patients who were also recruited as participants in the carriage study.

^b^Antimicrobial resistance phenotyping was conducted using Vitek2 software and interpreted using Clinical and Laboratory Standards Institute thresholds. All isolates were confirmed resistant to ampicillin. Additional drugs displaying acquired resistance are listed.

^c^Isolate identified as extended-spectrum β-lactamase producer.

### Genomic Diversity of *Klebsiella*

**Figure 1.**
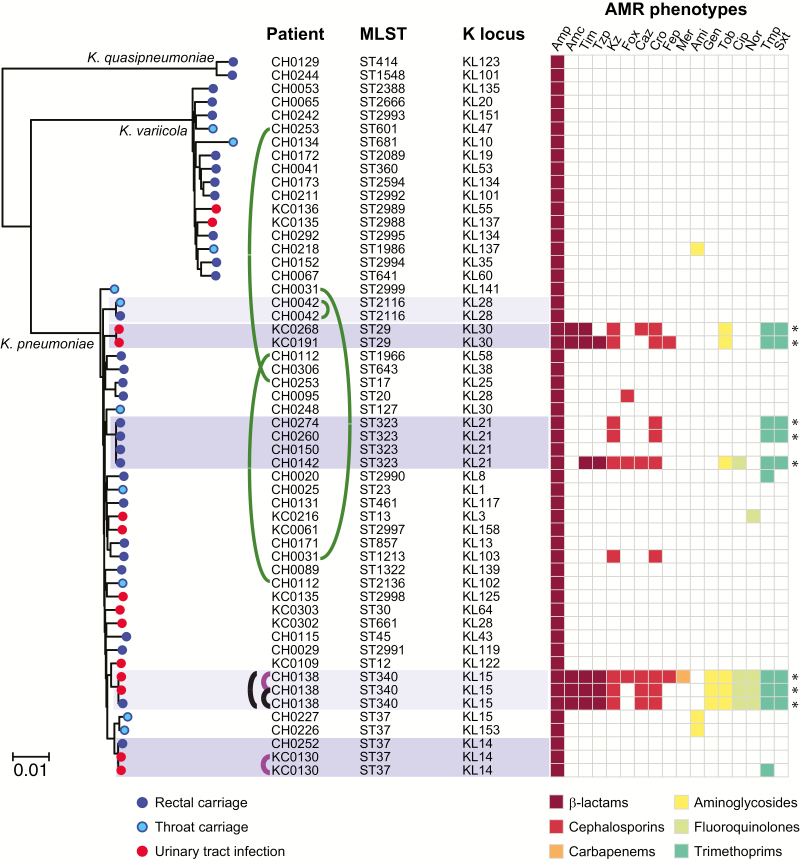
Core genome phylogeny for *Klebsiella* isolated from patients in the study wards, indicating multi-locus sequence typing (MLST) and capsule locus (KL) genotypes, and antimicrobial resistance (AMR) phenotypes. The tree is a maximum-likelihood phylogeny inferred from 489069 single-nucleotide variants in 3769 core genes; all branches defining species or lineages (ie, clades of isolates sharing sequence types from MLST) have >90% bootstrap support. Scale bar indicates nucleotide divergence. Clusters of genomes with patristic distance ≤0.1% nucleotide divergence are highlighted. Curved lines indicate isolates from the same patient (green, carriage isolates; purple, infection isolates; black, carriage and infection isolates). K loci were identified using Kaptive software. AMR phenotypes, determined using Vitek2 and interpreted according to Clinical and Laboratory Standards Institute guidelines, are indicated in the heat map and colored by drug class according to the inset legend; Asterisks denote multidrug resistance (resistant to ≥3 drug classes). Amc, amoxicillin–clavulanic acid; Ami, amikacin; Amp, ampicillin; Caz, ceftazidime; Cip, ciprofloxacin; Cro, ceftriaxone; Fep, cefepime; Fox, cefoxitin; Gen, gentamicin; Kz, cefazolin; Mer, meropenem; Nor, norfloxacin; Sxt, trimethoprim-sulfamethoxazole; Tim, ticarcillin–clavulanic acid; Tmp, trimethoprim; Tob, tobramycin; Tzp, tazobactam-piperacillin.

We sequenced the genomes of all 59 isolates identified as *K. pneumoniae* from patients in the study wards: 12 throat swab sample, 33 rectal swab sample, and 14 UTI isolates, originating from 53 patients. Five carriage isolate genomes were excluded from detailed phylogenetic analysis owing to sequence failure (n = 1) or mixed culture (n = 4). Genome data from the remaining 54 isolates confirmed they were members of the *K. pneumoniae* complex, which are typically indistinguishable biochemically [18]: 37 *K. pneumoniae,* 2 *Klebsiella quasipneumoniae*, 15 *Klebsiella variicola* (Figure 1). UTI isolates were predominantly *K. pneumoniae* (n = 12/14, 86%). Carriage isolates were more diverse, with only 63% *K. pneumoniae*, but the difference was not statistically significant (*P* = .20 using χ^2^ test).

A core genome tree of the 54 *Klebsiella* isolates (Figure 1) showed they represent a diverse population comprising 45 phylogenetically distinct lineages. These included some common MLST sequence types (STs) previously associated with AMR infections in hospitals (ST17, ST20, and ST340) [19] or severe community-acquired infections (ST23 and ST45) [18, 20], as well as 13 novel STs that were submitted to the MLST database for ST assignment (5 *K. pneumoniae* and 8 *K. variicola*). Thirty-seven distinct capsule locus types were also detected [10] (Figure 1 and Supplementary Table S1).

Of the 4 individuals with both rectal and throat isolates, 3 were colonized with different strains at each site (Figure 1). All *K. quasipneumoniae* and *K. variicola* isolates were singleton strains; that is, each represented a unique lineage detected in a single patient. In contrast, 3 *K. pneumoniae* lineages were identified in ≥2 patients and thus could potentially indicate transmission in CH (ST323, ST29, and ST37; dark shading in Figure 1). To investigate further, we used long-read sequencing to generate high-quality, completely resolved genome sequences for these isolates (Methods and Supplementary Table S3), and we calculated SNV distances between isolates within each cluster (Table 4). The ST37 isolates were separated by 1816 SNVs (433 mutations plus 1383 SNVs introduced by recombination; Supplementary Figure S1) and thus represent independent strains not linked by recent transmission. The ST323 and ST29 isolates were much more closely related (4–40 SNVs), consistent with recent transmission. These isolates were ESBL and are investigated in detail below.

**Table 4.** Pairwise Genetic Differences Between Isolates in Multipatient Lineages^a^

Patient ID by ST	Age, y	Sex	Sample ID	Specimen	Sample Date (dd/mm/yyyy)	SNVs, No.	
						Chromosome	FIB_K_/FII_K_ Plasmid
ST29							
KC0191	91	M	INF249	UTI	01/12/2013	…	…
CH0258	91	F	INF322	UTI	17/02/2014	4	39^b^
ST323							
CH0142	72	M	KSB1_4E	Rectal	10/09/2013	…	…
CH0150	85	M	KSB1_7E	Rectal	17/09/2013	4	NA
CH0260	72	F	KSB1_10J	Rectal	11/02/2014	19	0
CH0274	73	F	KSB2_1B	Rectal	25/02/2014	40	5
ST37 (KL14)							
CH0110	92	F	INF042	UTI	08/05/2013	…	…
CH0110	92	F	INF059	UTI	16/05/2013	4	NA
CH0252	85	M	KSB1_7J	Rectal	04/02/2014	1786	NA

Abbreviations: F, female; ID, identifier, M, male; NA, not available (no FIB_K_/FII_K_ sequence detected); SNVs, single-nucleotide variants; ST, sequence type; UTI, urinary tract infection.

^a^Each isolate within a lineage is listed with accompanying patient and sample data, as well as the number of chromosomal and plasmid SNVs detected between the isolate and the first collected isolate from the same lineage.

^b^In INF249, the FIB_K_/FII_K_ plasmid sequence is integrated into the chromosome.

### MDR Mechanisms and Transmission

Seven of the 54 isolates were MDR (Figure 1). These belonged to the 2 potential transmission clusters (ST29 and ST323) and UTI and colonizing isolates from patient CH0138 (ST340). To investigate the genetic mechanisms for resistance, we used long-read sequencing to completely resolve these genomes (Methods). The ST29 and ST323 MDR isolates each harbored 4–6 plasmids (Supplementary Table S3). Notably, all the acquired AMR genes in these genomes were localized to the same >200–kilobase pair FIB_K_/FII_K_ plasmid backbone encoded conjugative transfer functions. This shared close similarity (>90% coverage and 99% identity) with plasmid pKPN3-307_type A from an Italian *K. pneumoniae* [21]. However there were some differences in the MDR regions, resulting in different susceptibility profiles (Supplementary Figure S2).

Both CH ST29 UTI isolates displayed similar AMR profiles and harbored the same set of 12 AMR genes, including the ESBL gene *bla*_CTX-M-15_ (Supplementary Figure S2). In 1 of the ST29 isolates, INF322, the AMR genes were located on a 243634–base pair circular plasmid (pINF322), carrying the FIB_K_ and FII_K_ replicons. This entire plasmid sequence was integrated into the chromosome of INF249, within a 23S ribosomal RNA gene (Supplementary Figure S2A), flanked by copies of IS*26* and an 8–base pair target site duplication (GGCTTTTC). The pINF322 plasmid carried 8 copies of IS*26*, and the integration event in INF249 seems to have been mediated by the copy situated next to the *aac6-Ib* gene (Supplementary Figure S2A). The first ST323 carriage isolates, KSB1_4E, carried a plasmid sequence (pKSB1_4E) that differed from pINF322 by just 1 SNV. Two of the other 3 ST323 isolates carried the identical plasmid backbone to pKSB1_4E (no SNVs). All 3 harbored *bla*_CTX-M-15_ within a variable MDR region (Supplementary Figure S2B).

**Figure 2.**
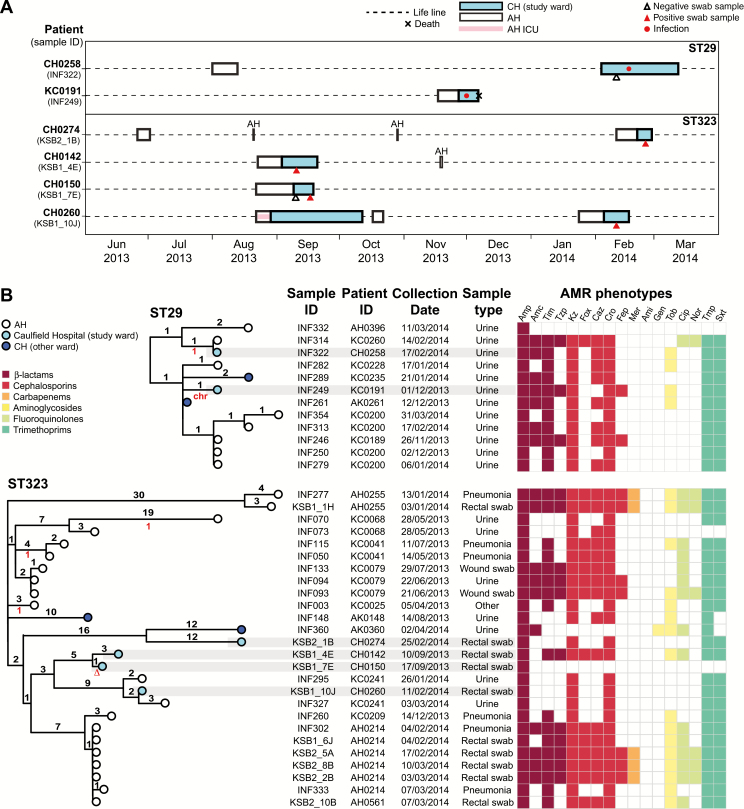
Multidrug-resistant extended-spectrum β-lactamase producing lineages associated with multiple patients in the study wards. *A,* Timelines for Caulfield Hospital (CH) and Alfred Hospital (AH) stays for patients in the CH study wards who were infected with sequence type (ST) 29 or colonized with ST323 *Klebsiella pneumoniae*. Details of all admissions to CH (study site) or AH (referring hospital) during the 1-year study period (1 April 2013 to 31 March 2014) were extracted from the hospital records of these patients. (Note that CH0274 and CH0142 short-term stays are all AH, not intensive care unit [ICU] or CH.) ID, identifier. *B,* Midpoint-rooted core genome phylogenetic trees for all ST29 and ST323 *K. pneumoniae* isolated at CH or AH during the study period. Tips are colored by hospital of corresponding specimen collection, according to inset legend. Isolates from the CH study wards are highlighted in gray. Branches are labeled with the number of chromosomal single-nucleotide variants (SNVs) defining the branch (black numbers, above branch) and the number of SNVs in the corresponding FIB_K_/FII_K_ plasmid sequence, relative to the major genotype (red numbers, below branch; see Supplementary Figure S2 for plasmid tree; ∆, plasmid lost; chr, plasmid integrated into chromosome). Dates of isolation are given in dd/mm/yyyy format. Antimicrobial resistance (AMR) phenotypes, determined using Vitek2 and interpreted according to Clinical and Laboratory Standards Institute guidelines, are indicated in the heat map and colored by drug class according to inset legend. Amc, amoxicillin–clavulanic acid; Ami, amikacin; Amp, ampicillin; Caz, ceftazidime; Cip, ciprofloxacin; Cro, ceftriaxone; Fep, cefepime; Fox, cefoxitin; Gen, gentamicin; Kz, cefazolin; Mer, meropenem; Nor, norfloxacin; Sxt, trimethoprim-sulfamethoxazole; Tim, ticarcillin–clavulanic acid; Tmp, trimethoprim; Tob, tobramycin; Tzp, tazobactam-piperacillin.

We investigated transmission of ST29 and ST323 strains and plasmids based on SNV counts, phylogenetic relationships, and hospital admission data (including time in CH and the referring hospital AH). In both the ST29 and ST323 clusters, the data did not support direct transmission between the patients in CH, because in many cases they lacked overlapping time in the wards (Figure 2A). Given the close genetic distances between isolates (4–40 SNVs, Table 4), we hypothesized that they may be linked by transmission in the referring hospital rather than CH. To investigate this we analyzed all ST29 and ST323 clinical isolates identified at AH and CH during the study period, and we constructed core genome phylogenies (Figure 2B). The CH ST29 isolates belonged to a group of 12 closely related strains (isolated in the last 4 months of the study) that shared a common ancestor with AH isolates, from which each differed by 1–4 SNVs (Figure 2B). Similarly, the CH ST323 isolates belonged to a group of 27 strains (isolated throughout the study) that shared a common ancestor with AH isolates, from which each differed by between 3 (early isolates) and 34 SNVs (late isolates).

All ST29 isolates and n = 26/27 ST323 isolates shared the same FIB_K_/FII_K_ plasmid sequence, separated by ≤1 SNV in the backbone sequence (note it is possible the remaining ST323 isolate also had the plasmid but lost it in culture). There was variation in the MDR regions, resulting in variable AMR phenotypes (Figure 2B and Supplementary Figure S2C), but *bla*_CTX-M-15_ was retained in all but 2 isolates.

### Carbapenem Resistance

**Figure 3.**
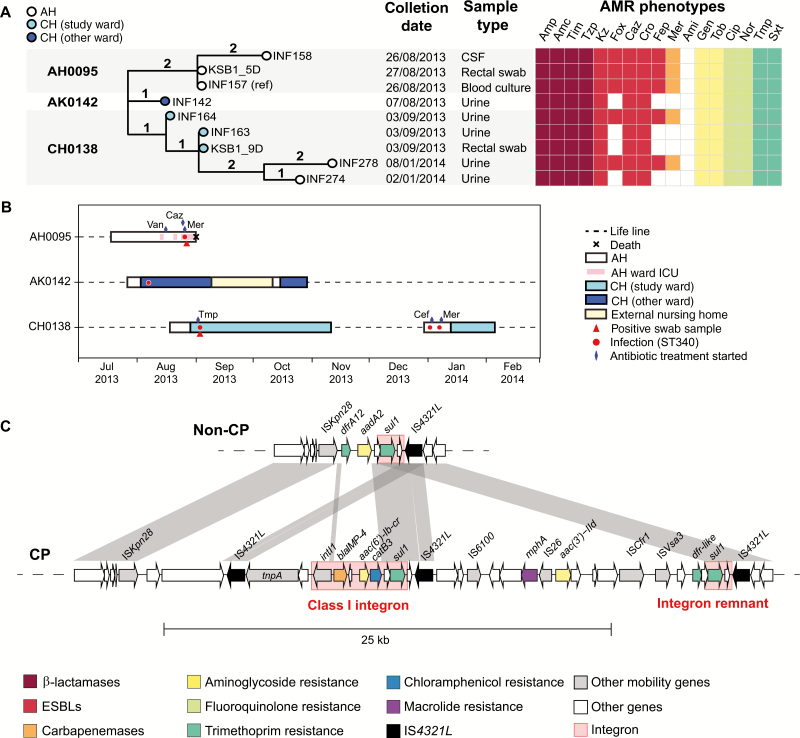
Carbapenemase producing lineage sequence type (ST) 340. *A,* Core genome phylogenetic tree of all ST340 isolates identified at Caulfield Hospital (CH) or Alfred Hospital (AH) during the study period. Branches are labeled with number of chromosomal single-nucleotide variants (SNVs). CSF, cerebrospinal fluid. Dates of isolation are given in dd/mm/yyyy format. Antimicrobial resistance (AMR) phenotypes, determined using Vitek2 and interpreted according to Clinical and Laboratory Standards Institute guidelines, are indicated in the heat map and colored by drug class according to inset legend. Amc, amoxicillin–clavulanic acid; Ami, amikacin; Amp, Ampicillin; Caz, ceftazidime; Cip, ciprofloxacin; Cro, ceftriaxone; Fep, cefepime; Fox, cefoxitin; Gen, gentamicin; Kz, cefazolin; Mer, meropenem; Nor, norfloxacin; Sxt, trimethoprim-sulfamethoxazole; Tim, ticarcillin-clavulanic acid; Tmp, trimethoprim; Tob, tobramycin; Tzp, tazobactam-piperacillin; *B,* Timelines for hospital stays; all admissions to CH (where study wards were located) or AH (referring hospital) during the 1-year study period (1 April 2013 to 31 March 2014) were extracted from hospital records of these patients. ICU, intensive care unit. *C,* Comparison of AMR regions in the 2 forms of IncC plasmid identified in these isolates; AMR genes and mobile elements genes are labeled and colored according to the inset legend; white arrows indicate genes of other or unknown functions. CP, carbapenemase producing, defined by presence of *bla*IMP-4 gene and phenotypic meropenem resistance (Mer in *A*); ESBLs, extended-spectrum β-lactamases.

Meropenem-resistant *K. pneumoniae* was detected in 1 CH patient (CH0138). Their rectal carriage isolate and 2 clinical urine isolates, collected on the same day, shared near-identical chromosomal sequences separated by a single SNV (Figure 3). Patient CH0138 was discharged after 2 months and readmitted to AH nearly 2 months later. *K. pneumoniae* was isolated from clinical urine samples on days 4 and 10 of the second admission. These were also ST340, separated from the earlier isolates by 3–5 SNVs (Figure 3).

Two distinct AMR patterns were observed among the CH0138 isolates: all were ESBL and MDR, however the first and last urine isolates were also resistant to cefoxitin, cefepime, and meropenem (Figure 3A). Completion of the genomes with long reads showed the MDR phenotypes were conferred by (1) chromosomal insertions of *sul1*, *aadA2* (via a type 1 integron) and *bla*_CTX-M-15_ (via IS*Ecp1*); (2) 2 large conjugative plasmids: a novel 90–kilo–base pair plasmid sharing 2 replicon genes (*repB* and *repE*) with pK245 (accession No. DQ449578), carrying *aac3-IIa, aac6Ib-cr, catB4, strAB, bla*_*OXA-1*_*, sul2* and *qnrB1*; and an IncC type 2 plasmid [16] (also known as IncA/C2 plasmid sequence type 3 [17]) carrying *bla*_*TEM*_*, aadA2, tetA, dfrA14* (Figure 3C and Supplementary Table S4). In the meropenem-resistant strains, the IncC plasmid also carried the carbapenemase *bla*_IMP-26_ as well as *mphA, aac3-IIa,* and *catB3* (Figure 3C).

Comparison with clinical isolates identified in AH during the study period identified 4 additional ST340 isolates from 2 patients, which shared the same plasmids and chromosomal AMR determinants and were separated from the first CH0138 isolate by 2–5 chromosomal SNVs (Figure 3A, Supplementary Table S4). Three strains (blood, cerebrospinal fluid, and rectal swab sample isolates) were isolated from patient AH0095 on admission to the ICU with sepsis. This followed a month-long stay in AH, which overlapped with the AH admissions of the other 2 patients, probably providing the opportunity for transmission (Figure 3B).

## DISCUSSION

We estimated overall carriage prevalence in the first week of admission to the geriatric unit at 13.5%, with 10.8% GI carriage and 4% throat carriage. GI carriage prevalence was significantly higher than we estimated previously for patients admitted to the AH ICU with no recent healthcare exposure [4] (5.9%; odds ratio [OR], 1.94 [95% CI, 1.0–3.7]; *P* = .03) but significantly lower than for ICU patients who had been in the hospital for >2 days or had undergone recent surgery (19%; OR 0.52 [0.3–0.9]; *P* = .02). The rate was also lower than that recently estimated for ICU and hematology/oncology patients at a US hospital [5] (23%; OR, 0.41 [95% CI, .27–.60]; *P* < .001).

Colonizing isolates were diverse (Figure 1) and mostly unique to individual participants, consistent with our earlier observations in ICU patients [4] and the recent US study [5]. Hence, in most cases, these strains probably represent established members of the patients’ microbiota rather than hospital-acquired bacteria. In the ICU, *K. pneumoniae* carriage is significantly associated with subsequent infection (OR, ≥4) [4, 5]. In the current setting, *K. pneumoniae* infections occurred at a similar rate (1.2% vs 1.8%–2.2%: *P* > .1) but the number of cases was too small to explore directly the link between colonization and infection. Notably, the 12 patients with *K. pneumoniae* infections were infected with 11 different lineages, consistent with most patients developing a UTI from their own microbiota. The exceptions were 2 UTIs associated with ESBL ST29, acquired by both patients in the referring hospital (Figure 2).

ESBL carriage (1.7%) was much more rare than previously reported in point prevalence studies of geriatric units or long-term care facilities [22, 23]. This is unsurprising, because we aimed to screen patients during the first week of their CH stay and focused only on *K. pneumoniae*. The MDR strains identified at CH all belonged to lineages that have been previously associated with hospital outbreaks of ESBL and/or carbapenemase-producing (CP) *K. pneumoniae* on other continents (ST29, ST323, and ST340) [24, 25], indicating the emergence of globally distributed ESBL strains in Australia. The ST258 strains harboring the *K. pneumoniae* carbapenemase, which are common internationally, have also been recently detected in Australia [26]. FIB_K_/FII_K_ plasmids are frequently reported as disseminators of *bla*_CTX-M-15_ and other AMR genes in *K. pneumoniae*, *Escherichia coli,* and other enteric bacteria isolated from humans, animals, and the environment [27, 28]. The IncC plasmid carrying *bla*_IMP_ was recently reported in *K. pneumoniae* (ST unknown) isolated from Australian wild birds [29], indicating that this plasmid and possibly the host strain are involved in spreading carbapenemases between animals and humans.

This study employed Illumina short-read whole-genome sequencing to identify high-confidence SNVs with which to identify lineages and AMR genes, bolstered by long-read sequencing to resolve plasmids and AMR gene context and maximize resolution for detecting transmission. Although this strategy has been used in other studies [30–33], ours is the first to adopt multiplex nanopore sequencing and hybrid assembly to rapidly and cost-efficiently complete genome sequences of interest in a high-throughput manner [11, 12] (Supplementary Tables S3 and S4). However, unravelling the source of MDR *K. pneumoniae* in CH also required exploring patient movement before CH admission, and phylogenetic context provided by additional isolates from the referring hospital.

Our analyses revealed that all MDR *K. pneumoniae* isolated at CH were linked to transmission clusters at the referring hospital (Figures 2 and 3). This suggests that MDR carriage is rare in the community, but MDR ESBL *K. pneumoniae* were occasionally acquired in the referring hospital before transfer to CH (ESBL GI carriage prevalence, 1.7%; ESBL infection incidence, 0.31%). These findings are likely generalizable to other hospital referral networks and highlight the benefits of exploring transmission at a multifacility level [34, 35]. However, larger studies will be needed to confirm the importance of referral networks for transmission of ESBL organisms, as has been demonstrated for MRSA and *Clostridium difficile* [34, 35], and to explore specific risk factors and the relevance to healthcare-associated infections.

It is noteworthy that the 2 ESBL lineages transmitting at AH (ST323 and ST29) shared the same FII_K_/FIB_K_*bla*_CTX-M-15_ plasmid. Given that ST323 seems to have been circulating at AH for months before the common ancestor of the ST29 strains, we hypothesize that the plasmid transferred from ST323 to ST29 within the hospital, promoting transmission of ST29 within AH. This highlights the importance of tracking ESBL or CP plasmids, as well as their host strains, as noted elsewhere [30, 31, 36].

The spread of CP *K. pneumoniae* within the referring hospital is concerning. The CP ST340 strain carrying *bla*_IMP_ and *bla*_CTX-M-15_ was identified in clinical isolates from 2 patients (patient AH0095, isolates KSB1_5D, INF157, and INF158; patient CH0138, isolate INF164). Additional variants lacking *bla*_IMP_ were found in 1 of these patients (patient CH0138) along with an ST340 UTI isolate from a third patient (patient AK0142) (Figure 3). Notably, the ST340 strain was detected on rectal swab samples from both patients who were screened for carriage (1 CH patient in this study; 1 ICU patient in the prior study [4]). However, it is encouraging to note that we detected no evidence of its transmission within the CH wards.

Only one-third of CH patients were recruited for GI carriage screening. Although 2 of the 3 patients with MDR infection were not screened, given the strong evidence that they acquired their infecting strain (ESBL ST29) in the referring hospital (Figure 2), it is possible they would have been detected as ESBL carriers if swab samples had been obtained on their arrival. Surveillance swab samples are frequently recommended to screen for carriage of MDR organisms in a variety of settings [37–39], but their role in the management of ESBL or CP gram-negative infections outside of outbreaks remains controversial [40]. Our study findings suggest that screening on transfer from tertiary referral hospitals to specialized hospitals could be valuable for the management or prevention of MDR infections.

## Supplementary Data

Supplementary materials are available at *Clinical Infectious Diseases* online. Consisting of data provided by the authors to benefit the reader, the posted materials are not copyedited and are the sole responsibility of the authors, so questions or comments should be addressed to the corresponding author.

Supplementary Methods

Supplementary Table 1

## Notes

***Acknowledgments.*** We gratefully acknowledge the contribution and support of Janine Roney, Mellissa Bryant, Jennifer Williams, Iain Abbott, and Noelene Browne at the Alfred Hospital, and the sequencing team at the Wellcome Trust Sanger Institute. We also thank the team of curators at the Institut Pasteur multilocus sequence typing platform (Paris, France) for importing novel alleles, profiles, and/or isolates at http://bigsdb.web.pasteur.fr.

***Financial support.*** This work was supported by the National Health and Medical Research Council of Australia (project 1043822 and fellowship 1061409 to K. E. H.) and the Australian Government Research Training Program (scholarship to C. L. G.).

***Potential conflicts of interest.*** All authors: No reported conflicts of interest. All authors have submitted the ICMJE Form for Disclosure of Potential Conflicts of Interest. Conflicts that the editors consider relevant to the content of the manuscript have been disclosed.
