# Supplementary material for: Antimicrobial-Resistant Klebsiella pneumoniae Carriage and Infection in Specialized Geriatric Care Wards Linked to Acquisition in the Referring Hospital
Source: Clin Infect Dis. 2018 Jan 11;67(2):161–70. doi: 10.1093/cid/ciy027 (PMC6030810; doi:10.1093/cid/ciy027)
Supplement: Supplementary Methods [file ciy027_suppl_supplementary_methods.pdf]

Supplementary Methods

Setting

The *Klebsiella* Acquisition Surveillance Project at Alfred Health (KASPAH) was conducted from April 1, 2013 to March 31, 2014 in Melbourne, Australia. Ethical approval for this study was granted by the Alfred Hospital Ethics Committee (Project number #550/12). Screening for *K. pneumoniae* rectal and throat carriage was conducted at the Alfred Hospital (a 350-bed tertiary referral hospital) and Caulfield Hospital (CH; a 230-bed hospital specializing in geriatric care and rehabilitation). Surveillance for clinical isolates from patients at CH and the Alfred Hospital, both of which are served by the Alfred Hospital Microbiology laboratory. Results of the intensive care component of KASPAH have been published previously[1]; the present report focuses on two geriatric wards (one acute and one sub-acute) at CH, which comprise mainly older patients admitted for rehabilitation or restorative care, treatment of various geriatric syndromes, or an acute medical problem. The majority of patients admitted to the geriatric wards were over 65 years old, however younger patients with similar clinical needs can also be admitted.

Recruitment into carriage study

Adult patients admitted to the two study wards were recruited into the screening study. Trained clinical research nurses visited the wards weekly to recruit participants. All adult patients aged ≥50 years present on the ward at the time of the visit, who had not been previously enrolled in the study (i.e. those who had been admitted within the week since the last visit), were eligible for inclusion. Verbal informed consent was required from the patient (or an adult responsible for the patient) to be enrolled in the study; there were no other exclusion criteria. Where potential participants were able to give informed consent (as determined by the medical staff on the ward), the study nurse approached them to explain the research project and to request verbal consent to participate; otherwise if an adult person responsible for the patient (e.g. next of kin) was present they were approached to provide verbal consent. Information explaining the project was provided to potential participants and/or their person responsible both verbally (by the nursing staff) and in the form of a written participant information sheet. Information sheets were made available at all times in the wards, and included contact information for the principal investigator and a statement that participants may withdraw at any time. Verbal consent was recorded using a pre-printed sticker indicating the acquisition of verbal consent, signed and dated by the study nurse and placed in the medical record. Where consent was provided by a next of kin, their name and relationship to the participant was recorded on the sticker.

Sample and patient data collection

Carriage swabs were collected during the weekly recruitment visits. One rectal swab and one throat swab were then taken and the date recorded. Accompanying patient data was collected for all study participants as soon as possible following recruitment. Each participant's medical reference number (MRN) was recorded and a study number assigned. A clinical questionnaire was then completed by nurses, based on current hospital records and charts.

This questionnaire included information on age, gender, date of hospital and ICU admission (if applicable), any surgery (including type) in the last 30 days, and any antibiotic treatment (including type given) in the last 7 days. For participants colonized or infected with MDR *K. pneumoniae* only, dates of discharge and/or death were extracted from hospital records at the conclusion of the study.

Screening swabs
Screening swabs were processed in the Alfred Hospital microbiological diagnostic laboratory as previously described[1]. Throat swabs were obtained using a sterile cotton swab, moistened with sterile normal saline, which was gently rolled across both palatal fauces with a mucosal contact time of 3-5 seconds. To obtain rectal swabs, a similar sterile cotton swab was moistened with sterile normal saline and then inserted into the distal rectum and gently rotated for 3 seconds.

Swabs were plated onto selective media and any with the appearance of *Klebsiella* species (convex, mucoid, pink on MacConkey agar) were investigated using matrix- assisted laser desorption ionization-time of flight (MALDI-TOF) analysis with a Vitek MS (bioMerieux, Marcy L'Etoile, France). If the colony chosen was not *K. pneumoniae,* two further colonies were sampled for MALDI-TOF testing. If a colony resembling *K. pneumoniae* had an unconfirmed identification reported by the MALDI-TOF, further identification analysis was performed using other methods as appropriate; these included motility testing and the Vitek2 GNI card (bioMerieux, Marcy L'Etoile, France).

The study design did not include collection of follow-up swabs, however ten participants had two swabs taken. In nine cases, the second swab was collected during a second hospital admission. The time lag between these samples was 14–209 days (median 46) and all were culture-negative for *K. pneumoniae*. One participant (CH0150) was swabbed twice, seven days apart, during a single admission; their first rectal swab (day 19) was culture-negative and the second rectal swab (day 26) was positive, yielding isolate KSB1_7E (both throat swabs were negative).

Clinical isolates
Clinical investigations at CH are conducted by referring specimens to the diagnostic service of the Alfred Hospital Microbiology laboratory. Clinical isolates were included in the present study when a treating physician at CH referred a specimen to the laboratory for analysis based on clinical suspicion of infection, and *K. pneumoniae* was subsequently identified and reported as a pathogen according to standard operating procedures. All samples identified in the two wards involved in this study resulted from urine samples; these were only reported as clinical isolates in the absence of other enteric bacteria.

Antimicrobial susceptibility testing
All clinical and carriage isolates were subjected to antimicrobial susceptibility testing, using the Vitek2 GNS card and CLSI breakpoints. Antimicrobials tested were: ampicillin, amoxicillin-clavulanate, ticarcillin-clavulanate, tazobactam-piperacillin, cefazolin, cefoxitin, cetazidime, ceftriaxone,

cefepime, amikacin, gentamicin, tobramycin, ciprofloxacin, norfloxacin, trimethoprim, and trimethoprim-sulfamethoxazole. If the susceptibility pattern suggested an extended- spectrum beta-lactamase this was confirmed using the method of Jarlier[2]. Isolates were assigned a binary phenotype (resistant or non-resistant, the latter including susceptible and insusceptible) according to CLSI breakpoints for each antimicrobial. Isolates were classified as multidrug resistant (MDR) if they were resistant to three or more classes of antibiotics.

DNA extraction and Illumina sequencing
DNA was extracted from overnight cultures, grown at 37°C in Luria-Bertani (LB) broth, using a phenol:chloroform protocol and phase lock gel tubes (5PRIME) and sequenced via Illumina HiSeq to generate 125 bp paired-end reads following preparations with either Nextera XT or TruSeq libraries (details in[1]). To construct the phylogeny in **Figure 1**, all read sets were aligned to the reference genome *K. pneumoniae* NTUH-K2044 (accession AP006725) and single nucleotide variants (SNVs) extracted using the RedDog pipeline (https://github.com/katholt/RedDog). A maximum likelihood phylogenetic tree was inferred from an alignment of all SNVs identified within 3769 core genes (present in all genomes) using FastTree v2.1.8[3] Lineages were defined from this tree using RAMI[4] with a patristic distance threshold of 0.01, and multi-locus sequence types (MLST) were assigned using SRST2[5].

Short read assembly and annotation
Illumina short reads were assembled with an in-house pipeline that undertakes read quality control (minimum Phred score of 30) using FastQC[6], before using SPAdes (v3.6.1)[7] to assemble the reads into contigs. Contigs were then ordered against the *K. pneumoniae* NTUH-K2044 reference genome with ABACAS and annotated, using the protein coding sequences from the same reference genome, with Prokka[8] Capsule locus types were identified from the resulting assembled contigs using Kaptive[9].

Long read sequencing and hybrid genome assembly
Isolates selected for long read sequencing and genome assembly were prepared and sequenced using a recently published protocol[10]. Briefly, DNA was extracted from overnight cultures, grown at 37°C in LB, using a modified version of Agencourt GenFind V2 (Beckman Coulter). Libraries were prepared without shearing, and using the Oxford Nanopore Technologies (ONT) 1D ligation sequencing kit (SQK-LSK10-8 and native barcoding expansion kit (EXP-NBD103). The final library was loaded onto an ONT MinION R9.4 flow cell and the run performed on a MinION MK1b device. Following basecalling of the long reads using Albacore (v1.1.2), the Illumina short reads and ONT long reads were subjected to hybrid assembly with Unicycler (v0.4.0)[11]. The resulting chromosome and plasmid sequences were annotated using Prokka[8], with the protein coding sequences from strain NTUH-K2044 as reference. Annotation of antimicrobial resistance regions were manually curated, with the help of the ARG-Annot database[12] and the Repository of Antibiotic resistance Cassettes (RAC) databases and annotation service[13,14], and submitted to GenBank. Accessions for Illumina reads, ONT reads and hybrid assemblies are given in **Table S1**.

High-resolution comparison of genomes belonging to the same lineage
Comparisons of isolates within lineages (ST29, ST37, ST323, ST340) were conducted as follows. The completed hybrid assembly of the earliest CH isolate from the lineage was selected as the reference for comparison. Using the RedDog pipeline (https://github.com/katholt/RedDog), Illumina reads were aligned to the reference chromosome to identify SNVs. Erroneous SNVs called when mapping the short reads corresponding to the reference were manually excluded. Gubbins[15] (v 2.0.0) was used to identify and exclude SNVs introduced into the lineage via recombination, and an alignment of the remaining SNVs was passed to RAxML (v8.2.9)[16] to infer a phylogenetic tree, with ascertainment bias correction and a general time reversible (GTR) model. No SNVs were identified and removed by Gubbins from the ST29, ST323 or ST340 lineages. Reference genomes were: ST29, INF322 (accession: CP024482); ST323, KSB1_4E (accession: CP024499); ST37: INF042 (accession: CP024542); ST340: INF157 (accession: CP024528). FIB_K/FII_K plasmid SNV analysis was conducted in the same manner, by mapping all ST323 and ST29 Illumina reads to pKSB1_4E (accession: CP024500); in this case Gubbins removed 74 of the 78 SNVs, leaving just 4 SNVs.

Statistical analysis
All statistical analyses were conducted using R (v3.3.1). The χ^2 test (function chisq.test) was used to investigate associations expressed in 2x2 contingency tables (two-sided tests in all cases). Logistic regression was used to test for the effect on *K. pneumoniae* carriage of age, days spent in hospital (prior to swab collection) and gender (**Table S2**), using the glm function.

Supplementary References

1. Gorrie CL, Mirčeta M, Wick RR, et al. Gastrointestinal carriage is a major reservoir of *Klebsiella pneumoniae* infection in intensive care patients. Clin Infect Dis **2017**; 65:208–215.

2. Jarlier V, Nicolas M-H, Fournier G, Philippon A. Extended Broad-Spectrum-Lactamases Conferring Transferable Resistance to Newer-Lactam Agents in Enterobacteriaceae: Hospital Prevalence and Susceptibility Patterns. Rev Infect Dis **1988**; 10:867–878.

3. Price MN, Dehal PS, Arkin AP. FastTree 2 – Approximately Maximum-Likelihood Trees for Large Alignments. PLoS ONE **2010**; 5:e9490.

4. Pommier T, Canbäck B, Lundberg P, Hagström Å, Tunlid A. RAMI: a tool for identification and characterization of phylogenetic clusters in microbial communities. Bioinformatics **2009**; 25:736–742.

5. Inouye M, Dashnow H, Raven L-A, et al. SRST2: Rapid genomic surveillance for public health and hospital microbiology labs. Genome

Med **2014**; 6:90.

6. Andrews S. FastQC: a quality control tool for high throughput sequence data. **2010**;

7. Bankevich A, Nurk S, Antipov D, et al. SPAdes: A new genome assembly algorithm and its applications to single-cell sequencing. Journal of Computational Biology **2012**; 19:455–477.

8. Seemann T. Prokka: rapid prokaryotic genome annotation. Bioinformatics **2014**; 30:2068–2069.

9. Wyres KL, Wick RR, Gorrie CL, et al. Identification of Klebsiella capsule synthesis loci from whole genome data. Microb Genom **2016**; 2.

10. Wick RR, Judd LM, Gorrie CL, Holt KE. Completing bacterial genome assemblies with multiplex MinION sequencing. Microb Genom **2017**; 3.

11. Wick RR, Judd LM, Gorrie CL, Holt KE. Unicycler: Resolving bacterial genome assemblies from short and long sequencing reads. PLoS Comput Biol **2017**; 13:e1005595.

12. Gupta SK, Padmanabhan BR, Diene SM, et al. ARG-ANNOT, a new bioinformatic tool to discover antibiotic resistance genes in bacterial genomes. Antimicrob Agents Chemother **2014**; 58:212–220.

13. Tsafnat G, Coiera E, Partridge SR, Schaeffer J, Iredell JR. Context-driven discovery of gene cassettes in mobile integrons using a computational grammar. BMC Bioinformatics **2009**; 10:281.

14. Tsafnat G, Copty J, Partridge SR. RAC: repository of antibiotic resistance cassettes. Database **2011**; 2011.

15. Croucher NJ, Page AJ, Connor TR, et al. Rapid phylogenetic analysis of large samples of recombinant bacterial whole genome sequences using Gubbins. Nucleic Acids Research **2015**; 43:e15–e15.

16. Stamatakis A. RAxML version 8: a tool for phylogenetic analysis and post-analysis of large phylogenies. Bioinformatics **2014**; 30:1312–1313.

Supplementary Tables

Table S1. Full details of isolates used in this study.
Fields include: sample ID, specimen type, specimen collection date, multi-locus sequence type (ST), capsule locus type (KL), hospital in which sample was collected (and ward if relevant to CH study), AMR phenotypes (if isolate showed resistance to drugs tested, not including ampicillin), acquired AMR genes, and accession numbers for all sequence data in NCBI.

[see separate document ‘SupplementaryTable1.txt’]

Table S2. Logistic regression models of *K. pneumoniae* carriage. Values given are odds ratio [95% confidence interval] and p-value.

	**Unadjusted (One model per variable)**	**Adjusted (All variables in one model)**
**Age (in years)**	0.97 [0.93 – 1.01], p=0.09	0.97 [0.94 – 1.01], p=0.15
**Days in hospital (current admission)**	1.01 [0.97 – 1.04], p=0.53	1.01 [0.97 – 1.03], p=0.85
**Female Sex**	1.51 [0.77 – 3.00], p=0.23	1.39 [0.70 – 2.79], p=0.34

Table S3. Genome information for isolates belonging to multi-patient lineages. Genomes were finished using hybrid assembly of short (Illumina) and long (Oxford Nanopore) reads. Plasmid replicon types and acquired AMR genes were identified via BLAST search (coverage ≥90%, identity ≥90%) of the finished genomes using PlasmidFinder and ARG-Annot databases. Accessions are given in Table S1.

Sample ID (Patient ID)	Replicon sizes (bp)		Acquired AMR genes
	**Chromosome**	**Plasmid (rep type)**	
**ST29**			
**INF249 (KC0191)**	5,583,461	71,140 (FIAHI1)<br>6,657<br>5,783<br>3,514 (Col)<br>3,223<br>2,273 (Col)	Chromosome (integrated FIB$_K$/FII$_K$ plasmid backbone):<br>*sul2, strAB, bla$_{TEM-1}$, bla$_{CTX-M-15}$, catB4* (partial), *bla$_{OXA-1}$, aac6Ib-cr, tetA, qnrB1, dfrA14*
**INF322 (CH0258)**	5,356,173	243,634 (FIB$_K$/FII$_K$)<br>71,104 (FIAHI1)<br>4,228 (Col)<br>3,514 (Col)<br>3,223<br>2,273 (Col)	FIB$_K$/FII$_K$ plasmid:<br>*sul2, strAB, bla$_{TEM-1}$, bla$_{CTX-M-15}$, catB4* (partial), *bla$_{OXA-1}$, aac6Ib-cr, tetA, qnrB1, dfrA14*
**ST323**			
**KSB1_4E (CH0142)**	5,234,963	243,620 (FIB$_K$/FII$_K$)<br>212,195 (FIB)<br>4,064<br>3,223	FIB$_K$/FII$_K$ plasmid:<br>*sul2, strAB, bla$_{TEM-1}$, bla$_{CTX-M-15}$, catB4* (partial), *bla$_{OXA-1}$, aac6Ib-cr, tetA, qnrB1, dfrA14*
**KSB1_7E (CH0150)**	5,232,513	212,097 (FIB)<br>4,064	-
**KSB1_10J (CH0260)**	5,228,900	227,807 (FIB$_K$/FII$_K$)<br>212,079 (FIB)<br>4,064<br>3,223<br>1,916 (Col)	FIB$_K$/FII$_K$ plasmid:<br>*sul2, strAB, bla$_{TEM-1}$, bla$_{CTX-M-15}$, catB4* (partial), *dfrA14, qnrB1*
**KSB2_1B (CH0274)**	5,228,889	310,025 (FIB)<br>228,353 (FIB$_K$/FII$_K$)<br>140,704 (FII)<br>98,344 (FII$_K$)<br>3,514 (FCol)<br>3,377<br>3,336<br>3,223<br>3,012<br>2,936	FIB$_K$/FII$_K$ plasmid:<br>*sul2, strAB, bla$_{TEM-1}$, bla$_{CTX-M-15}$, catB4* (partial), *dfrA14*
**ST37**			
**INF042 (CH0110)**	5,337,491	110,374 (FII/FIAHI1)<br>71,587 (FIIp14)	-
**INF059 (CH0110)**	5,337,491	110,374 (FII/FIAHI1)<br>71,587 (FIIp14)	-
**KSB1_7J (CH0252)**	5,354,543	-	-

Table S4. ST340 samples are listed, from patients exhibiting carbapenem resistant carriage or infection isolates. Size of chromosome for each isolate is given, along with plasmid details including: size of plasmids; plasmid incompatibility (Inc) types, as well as plasmid sequence type (pST), if identifiable; and any resistance genes in the genome.

Sample ID (Patient ID)	Replicon sizes (bp)		Resistance genes
	Chromosome	Plasmid (rep type)	
**KSB1_9D (CH0138)**	5,264,380	187,611 (C2 (pST3), P)<br>147,932 (FIB_K, FII_K)<br>89,345 (FIAHI1, R)<br>4,660<br>4,510<br>3,825 (Col)	Chromosome: *aadA2, ampH, $bla_{CTX-M-15}$, sul1*<br>IncC plasmid: *bla_{TEM}, aadA2, tetA, dfrA14*<br>FIAHI1/R plasmid: *aac3-IIa, strA, strB, bla_{OXA-1}, sul2, qnrB1, aac6Ib-cr, catB4*
**INF163 (CH0138)**	5,298,228	187,611 (C2 (pST3), P)<br>147,932 (FIB_K, FII_K)<br>89,345 (FIAHI1, R)<br>4,660<br>4,510<br>3,825 (Col)	Chromosome: *aadA2, ampH, $bla_{CTX-M-15}$, sul1*<br>IncC plasmid: *bla_{TEM}, aadA2, tetA, dfrA14*<br>FIAHI1/R plasmid: *aac3-IIa, strA, strB, bla_{OXA-1}, sul2, qnrB1, aac6Ib-cr, catB4*
**INF164 (CH0138)**	5,298,459	217,685 (C2 (pST3), P)<br>147,945 (FIB_K, FII_K)<br>89,345 (FIAHI1, R)<br>4,660<br>4,510<br>3,825 (Col)	Chromosome: *aadA2, ampH, $bla_{CTX-M-15}$, sul1*<br>IncC plasmid: *bla_{TEM}, aadA2, tetA, dfrA14, mphA, aac3-IIa, catB3, bla_{IMP-26}*<br>FIAHI1/R plasmid: *aac3-IIa, strA, strB, bla_{OXA-1}, sul2, qnrB1, aac6Ib-cr, catB4*
**INF274 (CH0138)**	5,293,333	187,611 (C2 (pST3), P)<br>147,932 (FIB_K, FII_K)<br>90,173 (FIAHI1, R)<br>4,660<br>4,510<br>3,825 (Col)	Chromosome: *aadA2, ampH, $bla_{CTX-M-15}$, sul1*<br>IncC plasmid: *bla_{TEM}, aadA2, tetA, dfrA14*<br>FIAHI1/R plasmid: *aac3-IIa, strA, strB, bla_{OXA-1}, sul2, qnrB1, aac6Ib-cr, catB4*
**INF278 (CH0138)**	5,293,333	187,611 (C2 (pST3), P)<br>147,932 (FIB_K, FII_K)<br>89,345 (FIAHI1, R)<br>4,660<br>4,510<br>3,825 (Col)	Chromosome: *aadA2, ampH, $bla_{CTX-M-15}$, sul1*<br>IncC plasmid: *bla_{TEM}, aadA2, tetA, dfrA14*<br>FIAHI1/R plasmid: *aac3-IIa, strA, strB, bla_{OXA-1}, sul2, qnrB1, aac6Ib-cr, catB4*
**INF157 (AH0095)**	5,297,108	221,606 (C2 (pST3), P)<br>147,932 (FIB_K, FII_K)<br>89,345 (FIAHI1, R)<br>4,660<br>4,510<br>3,825 (Col)	Chromosome: *aadA2, ampH, $bla_{CTX-M-15}$, sul1*<br>IncC plasmid: *bla_{TEM}, aadA2, tetA, dfrA14, mphA, aac3-IIa, catB3, bla_{IMP-26}*<br>FIAHI1/R plasmid: *aac3-IIa, strA, strB, bla_{OXA-1}, sul2, qnrB1, aac6Ib-cr, catB4*
**INF158 (AH0095)**	5,297,118	221,606 (C2 (pST3), P)<br>147,932 (FIB_K, FII_K)<br>89,345 (FIAHI1, R)<br>4,660<br>4,510<br>3,825 (Col)	Chromosome: *aadA2, ampH, $bla_{CTX-M-15}$, sul1*<br>IncC plasmid: *bla_{TEM}, aadA2, tetA, dfrA14, mphA, aac3-IIa, catB3, bla_{IMP-26}*<br>FIAHI1/R plasmid: *aac3-IIa, strA, strB, bla_{OXA-1}, sul2, qnrB1, aac6Ib-cr, catB4*
**KSB1_5D (AH0095)**	5,296,999	221,606 (C2 (pST3), P)<br>147,945 (FIB_K, FII_K)<br>89,345 (FIAHI1, R)<br>4,660<br>4,510<br>3,825 (Col)	Chromosome: *aadA2, ampH, $bla_{CTX-M-15}$, sul1*<br>IncC plasmid: *bla_{TEM}, aadA2, tetA, dfrA14, mphA, aac3-IIa, catB3, bla_{IMP-26}*<br>FIAHI1/R plasmid: *aac3-IIa, strA, strB, bla_{OXA-1}, sul2, qnrB1, aac6Ib-cr, catB4*

Supplementary Figures

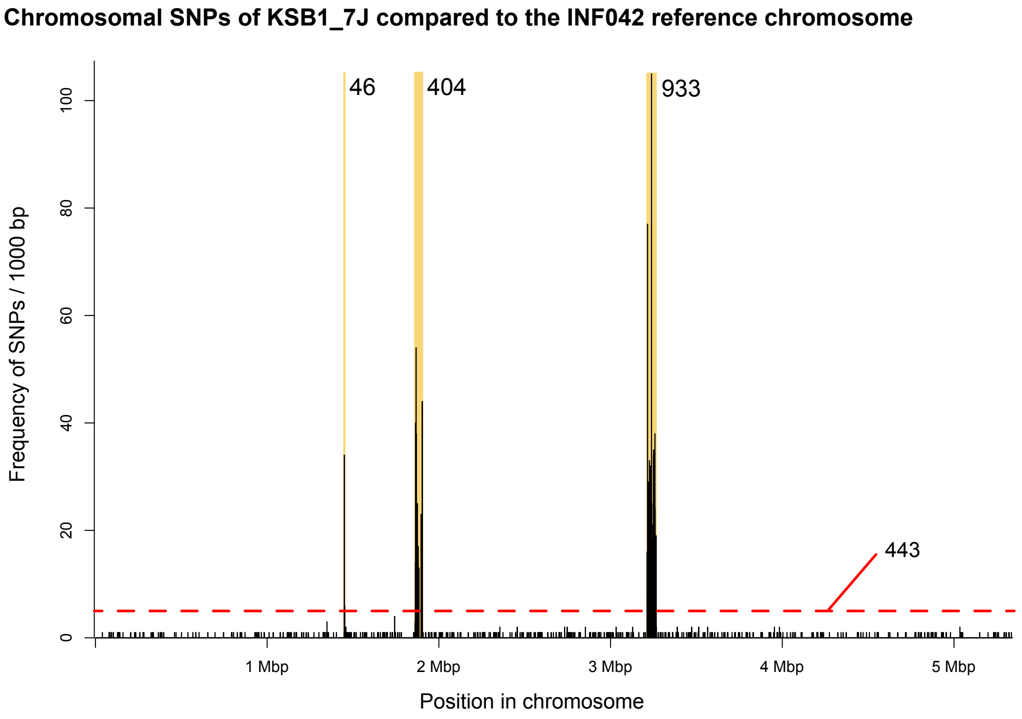

Figure S1. Distribution of single nucleotide variants (SNVs) between ST37 KL14 genomes. SNVs were identified by mapping short reads from KSB1_7J (patient CH0252) to the finished genome sequence of INF042 (patient CH0110). Regions with dense clusters of SNVs, presumed to be introduced via homologous recombination, are coloured in yellow and labeled with the total number of SNVs in each region. Dashed red line indicates a frequency of 5 SNVs per 1,000 bp; a total of 443 SNPs fall below this line, excluding those in the recombinant regions.

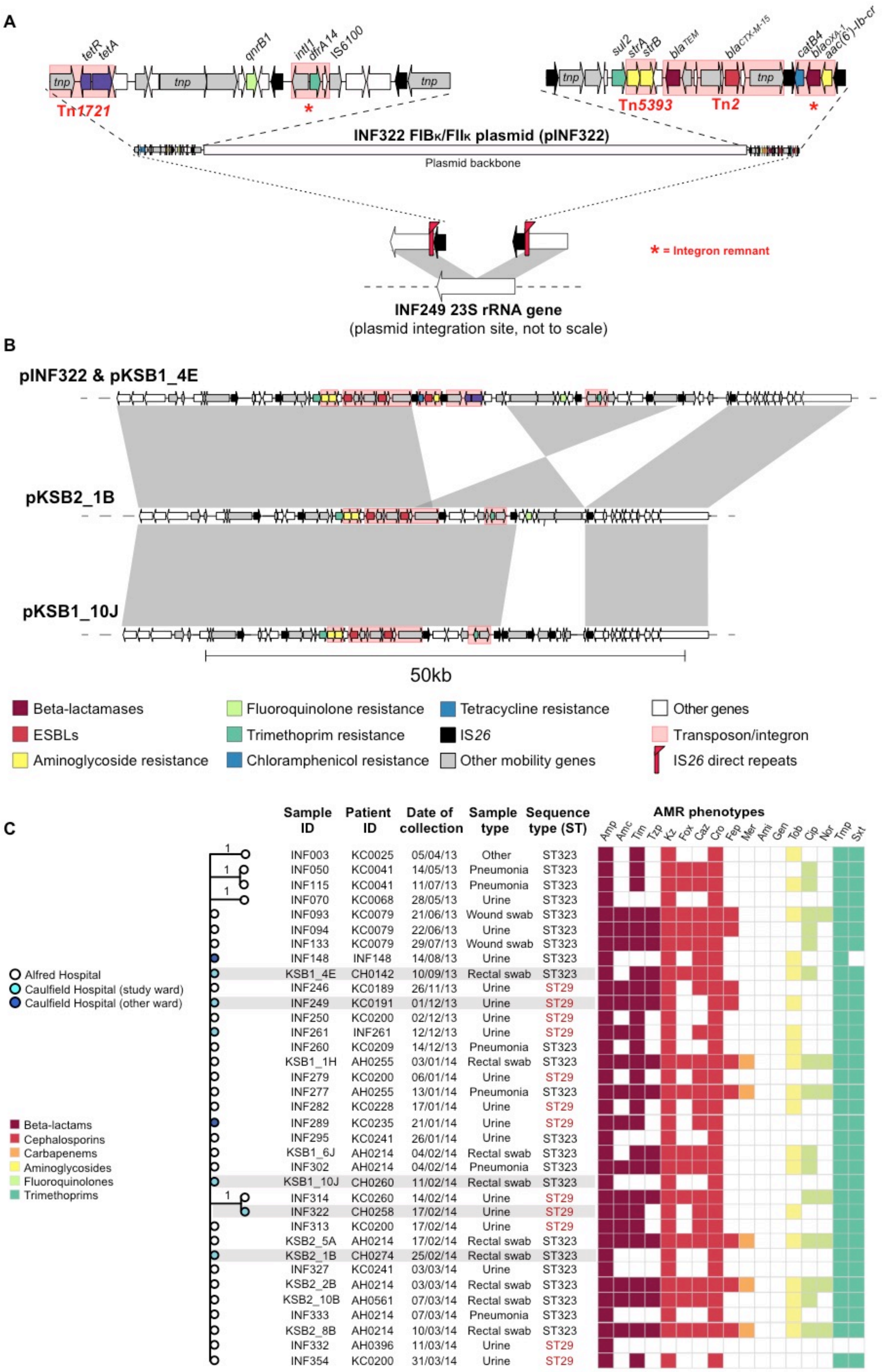

Figure S2. FIB$_K$/FII$_K$ plasmid diversity in ST29 and ST323 isolates.

(A) Genetic structure of the drug resistance region, and insertion site of the pINF322 plasmid sequence into the INF249 chromosome. Antimicrobial resistance (AMR) and transposase genes are labeled and coloured according to inset legend.
(B) Comparison of three different variants of the drug resistance region identified amongst FIB_K/FII_K plasmids in ST29 and ST323 isolates from CH patients. AMR and transposase genes are coloured as in panel **A**, according to inset legend.
(C) Phylogenetic tree of the FIB_K/FII_K plasmid detected in ST29 and ST323 isolates at CH and Alfred Hospital. Tree is based on single nucleotide variants (SNVs) detected in the plasmid backbone, identified by mapping short reads from all ST29 and ST323 isolates to the pKSB1_4E plasmid sequence; branches are annotated with their length (SNV count). Dates of isolation are given in dd/mm/yyyy format. AMR phenotypes, determined using Vitek2 and interpreted according to CLSI guidelines, are indicated in the heatmap and coloured by drug class according to inset legend. Amp, Ampicillin; Amc, Amoxicillin-clavulanic acid; Tim, Ticarcillin-clavulanic acid; Tzp, Tazobactam-piperacillin; Kz, Cefazolin; Fox, Cefoxitin; Caz, Ceftazidime; Cro, Ceftriaxone; Fep, Cefepime; Mer, Meropenem; Ami, Amikacin; Gen, Gentamicin; Tob, Tobramycin; Cip, Ciprofloxacin; Nor, Norfloxacin; Tmp, Trimethoprim; Sxt, Trimethoprim-sulfamethoxazole.
